# Supplementary material for: A PPR Protein RFCD1 Affects Chloroplast Gene Expression and Chloroplast Development in Arabidopsis
Source: Plants (Basel). 2025 Mar 15;14(6):921. doi: 10.3390/plants14060921 (PMC11944589; doi:10.3390/plants14060921)
Supplement: Supplementary file 1 [file plants-14-00921-s001.zip › Supplementary information.pdf]

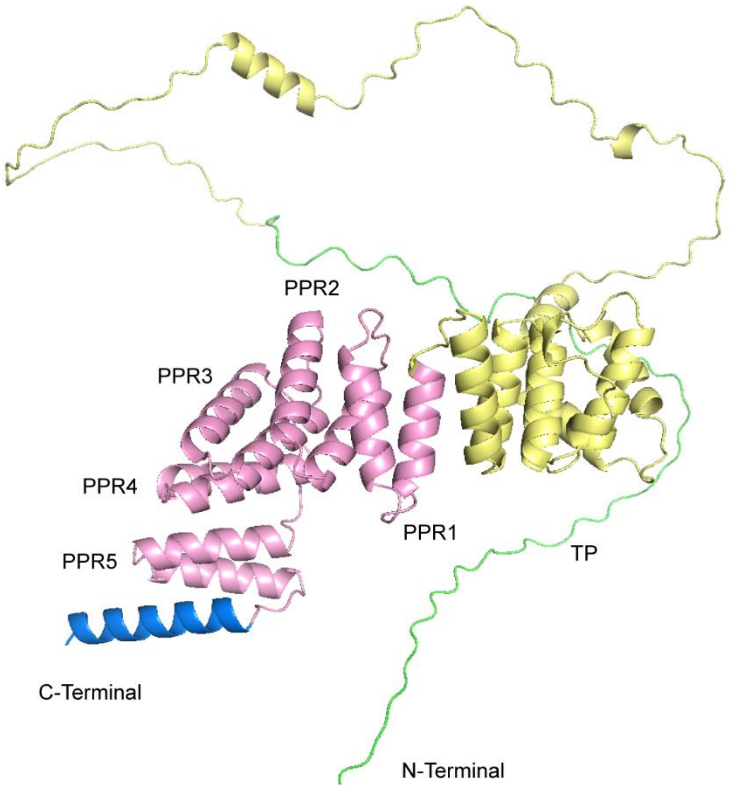

Supplementary Figure S1. Protein structure analysis of RFCD1. 3D structure of the RFCD1 protein predicted by Alphafold2 showing the arrangement of the PPR repeats. The N-terminal region (residues 1-60) is highlighted in green and corresponds to the chloroplast transit peptide. The PPR repeats are colored in pink.

```
                          *        20         *        40         *        60
A. thaliana     : -------------------------------------------------------MILHC :   5
P. trichocarpa  : -----------------------------------------------------MSKAMDW :   7
G. raimondii    : --------------------------MPVSEKFPLGIGASPFLCTRFILQTNDNTKKMDC :  34
G. max          : ---------------------------------------------------------MDC :   3
V. vinifera     : --------------------------------------------MERLFSSLSICTVPGT :  16
Z. mays         : --------------------------------------------------MHPRCPVLDF :  10
O. sativa       : ------------------------------------------------------------ :   -
A. coerulea     : --------------------------------------------MDSVLIT--CYKLHTM :  14
A. trichopoda   : ----------------------------------------------MASFLIPNQVILRA :  14
T. plicata      : ----------------------------------------------MMCILSENWAYSGL :  14
C. richardii    : ------------------------------------------------------------ :   -
D. complanatum  : -------------------------------------------------------MADAL :   5
M. polymorpa    : MDKWAWKLQKGALSRTVKDLGRMQLTEKVLPIFLWLQRQPHLWPDEITLCAAIHVMVEAG :  60

                          *        80         *       100         *       120
A. thaliana     : PVSLSLSFHLNLRTSRIGNIG--VTRVNASQRNHSKKLTKNLRNPRRTKLPPDFGVN--- :  60
P. trichocarpa  : KLTSISLHPILPTASNVLKSSSMILMAHSSTRICYRKIPKNIRYPRRSKLPPDFGVN--- :  64
G. raimondii    : IFSSITTYPFITLRGKLQQSICVTNLAHPNTRICYSKQPKNRRYPRRTKLPPDFGVN--- :  91
G. max          : TVSFPCVH-FVPVGRTLCNYRRVTVVAHSKR----RRPPKDNRYRPRPKQPPEFGVN--- :  55
V. vinifera     : RIYWDKRYVKAQFVVEFSNKNEIKLGVQCNARVNHRKPTKNLPHPRRAKLPPEPEIS--- :  73
Z. mays         : PLPMPEMKAATALGSWLLPAAPAIRPTLVNLRSRGFCSHGYFPSASRISCSSELSDS--- :  67
O. sativa       : ----------MSLVNRPLPALYGICTASAKTRAWWLCRDGNLPSTSRISCTEPSNGG--- :  47
A. coerulea     : VEIQKSQFKISITTISLEKRSKIIVMAGSNSGNSQRKLPKNLRYPRRAKVPVDIKAN--- :  71
A. trichopoda   : GIKLVNQQFISIGYCRYGENSCFRIQMMATK---HKKKPKNLRYPRRQKLPVDPALYKIQ :  71
T. plicata      : AISRGIEVPHNNLLKYNLGCRQFNRSKFGKMPWVSLIETSKAR-ICTIKVNSSIQVDG-- :  71
C. richardii    : ------------------------------------------------------------ :   -
D. complanatum  : LLSLCMNVVPHHLVAKPQNNVFVFRGRRSDVASNMECSP--------------------- :  44
M. polymorpa    : EIGVAMQLHRVKILARAEEGAMDALGRNLGLCLSTSVSSSTKRIRSVSEFSGEASVDF-- : 118

                          *       140         *       160         *       180
A. thaliana     : ---------LFLRKP-----------KIEPLVIDDDD--EQ-------------VQESVN :  85
P. trichocarpa  : ---------LFLKKP-----------QTDSVQDHSDDLTEE------------EEEEEIE :  92
G. raimondii    : ---------LFLEKPRTETDMSTDTEQIKSIYGHNNDSPEE------------EDDKEDH : 130
G. max          : ---------LFLKKP-----------STASKPTDDDMDSNE------------ENDEEED :  83
V. vinifera     : ---------TFLKGGNSGTEQS----EMGTVLDKEPDANDDG----------FLVDGIEG : 110
Z. mays         : ---------DRGLAK-----------EMES--EFSDEICAEN-------------GAEQD :  92
O. sativa       : ---------SVMELE-----------VMDRNEQTYHENSSAS-------------EDEDD :  74
A. coerulea     : ---------LYYKKKNIGID------DNERFQSFGGNNVEN-------------DEEEED : 103
A. trichopoda   : PAICDESSISFEQGVGLERGNSPNPNELEAISSLYQDEVRRKPRTLGKETNISYERGEEG : 131
T. plicata      : ----------FQAEEH----------AMSEMSGTWDEEMNH-------------ISDGDS :  98
C. richardii    : -------------------------------------------------------MEMQG :   5
D. complanatum  : ---------LAEEQRDAFAQKTASEDGEGKFVFPFS------------------------ :  71
M. polymorpa    : -----KKFVLGLRERDVVCYGSGRPDRRGRRDLPYIRKISRDRQKERERREAGIAEEEDD : 173
```

```
                                    *         200         *         220         *         240
A. thaliana      : DDDDAVVWEPEEIEAISSLFQKRIP-----------QKPDKPS---RVRPLPLPQPHKLR : 131
P. trichocarpa   : VNNGEIVWESEEIEAISSLFRGRIP-----------QKPGKLG---RERPLPLPVPYKLR : 138
G. raimondii     : VN-IDLAWESDEVEAISSLFQGRIP-----------QKPGKVG---RERPLPVPLPYKLR : 175
G. max           : GN-IGVVWESDELEAISSLFQGRIP-----------QKPGKLD---RERPLPLPVPFKLR : 128
V. vinifera      : RKEGEIVWDSDEIEAISSLFMGRIP-----------QKPGKLN---RERPLPLPLPYKLR : 156
Z. mays          : DETEDLIWSKEEIDAISALFDRPMR-----------QKPLKPRNPARQRALPLPLPHKTR : 141
O. sativa        : DEEEAVEWSKDELDAISALFDRPMR-----------QKPPKPPNPVRQRPLPLPLPHKTR : 123
A. coerulea      : DDDGEVKWSTDELETISSLFQGRIP-----------QKPGNLN---KERPLPLPIPYTIR : 149
A. trichopoda    : VERHEDSWSPDELDTIFSLFQGRIP-----------QKPGKLG---RVRPLPLPTPHKLR : 177
T. plicata       : IEKNEEEWSADQLDAIASLFRSPTP-----------TKSRSQM---KERYLPLPMPHKIR : 144
C. richardii     : KLGTQEVWTAEEWEIVKALLRTRPK-----------PFPLDVR------PLPPRVQSTYR :  48
D. complanatum   : PFEGSSFRSMNAEAVEMEQKERRLP------------LPMPHK---LARPAALPRSKHIL : 116
M. polymorpha    : AFEESDELRSGGEKIGKLPIESNGPPLLILHPYISDEKPVDER---FLRPLPLPSPHKLR : 230

                                    *         260         *         280         *         300
A. thaliana      : PLGLPTPKKNIIRS-----PALSSVSKQVYKDPSFLIGLAREIKSLPSSDADVSLVLNKW : 186
P. trichocarpa   : PLGLPAPKKHVNKQ---VSLSRASISSQIYKNPSFLIGLAKEIKRLSP-DQDVSVVLDNC : 194
G. raimondii     : PLGFPMPKKHVKKSSAGVNSSRASVSQQLYKNPSFLIGLSKEIKDLAS-DDDVSAVLNKW : 234
G. max           : PLRLPTPKTQVKLTAPAVVSSRASMAKKVYKSPSFLVGLARQISRLGP-DADVSKILGKW : 187
V. vinifera      : PMGLPTTKRHVRAASSMPYASRASLSKQVYKNPDFLISIAREIRKLPL-EDDVSPVLNKW : 215
Z. mays          : LPVAPAPKQHVRLAARAGLSSRACFSDQVRKKPEFLLGIAREIAALPP-EHDVSTVLDRW : 200
O. sativa        : LPNAPAPKQHIRLAARAALSSRSSFSDQVCKNPEVLIGIAREIAALPP-ESDVSIVLDRW : 182
A. coerulea      : PLGFPTSKKHVRRVS----PQRKTVCDKVYKNPTFLVHLAKEIKDLPA-EKDVSEVLNKW : 204
A. trichopoda    : PLGLPSPKNHIRSAYPAITASRSSLCKLNYKNPDFLLEIAREIRGLPP-EKNSSEVLNKR : 236
T. plicata       : PTGVPQSKRAFRSP---------NPLNRVYKNPQFLINLAREIRYLPP-GEEVSKVLDKW : 194
C. richardii     : PISVPTRRLRLTNR-----------KNSSAKDKHFLTSLAVQIASLPP-QTNASHILVGH :  96
D. complanatum   : RSRFPTWKPYVSGH-------MSHADRG-------LEKLAAEIHDLPP-ATPAAKVLAAW : 161
M. polymorpha    : PLRLPKPSLRRKVR-------IAVSTRDRVQSFDFLKDLASEIRSQPD-DEPLSELMDKW : 282

                                    *         320         *         340         *         360
A. thaliana      : VSFLRKGSLSTTIRELGHMGLPERALQTYHWAEKH--SHLVPDNRILASTIQVLAKHHEL : 244
P. trichocarpa   : SRYLHKGSLSLTIRELGHLGLPERALQTFCWVQKQ--PRLFPDDRVLASTVEVLARNHDL : 252
G. raimondii     : APFLRNGSLSITIRELGLMDLPQRALQAFCWAQKM--PHLYPDDRILTSTVEVLARKREL : 292
G. max           : VQFLRKGSLSLTIRELGHMGFPERALQTFLWAQNQ--PHLFPDDWILASTVEVLARNHEL : 245
V. vinifera      : VRFLRKGSLSLTIRELGHMGLPERALQTFFWAQKQ--PQLFPDDRILASTVEVLARTHKL : 273
Z. mays          : ARFLRKGSLSLTIRELGHMGLPERALQTLCWAQRQKAVPLFPDDRVLASAIEVLARFERL : 260
O. sativa        : VRFLRKGSLSMTIRELGHMGLPERALQTLCWAQRQTVVPLFPDDRILASTIEVLARFDQL : 242
A. coerulea      : VRFLRKGSLSLTIRELGHMNLPERALQTFSWAEKH--PHLFPDDRILGSTVEILARTRQM : 262
A. trichopoda    : YRILRKGSLSLTIRELGHMGLPHRALETFCWAQRH--PHLFPDDRLLASTVEVLARTGRL : 294
T. plicata       : DFVLRKGSLSLTIRELGHMGLPQRALQTFCWAQKC--PKLYPDDRVLGSTLQVLARAGEL : 252
C. richardii     : KHQLIRGSLSATIRELGHLGLACRALETFQWQQCR--HLWPDERTLCATIEVLTAGGKE : 154
D. complanatum   : ERYLKRGSLSLIIRELGRMRLSFRVLQVISWLQGI--PRLWPDEHSLCAAIKALVDAGEV : 219
M. polymorpha    : AWKLQKGALSRTVKDLGRMQLTEKVLPIFLWLQRQ--PHLWPDEITLCAAIHVMVEAGEI : 340
```

```
                      *        380          *        400          *        420
A. thaliana     : KL---LKFDNSLASKNVIEAMIKGCIEGGWLNLARKLILISKSNNRILDSSVYVKMILEI : 301
P. trichocarpa  : KVPFNLEKFTNLASRRVIEAMVKGLIRGGSLKLAWKLISVAKDGKRMLDPSVYAKIILEL : 312
G. raimondii    : KLPVNLEKFTSSTNRSLIEAMLKGFVKGGSLNLAWKLLSVLKQSKRMLDPGIYAKLILEL : 352
G. max          : RIPFNLGQYTGLASRAVLEAMIKGFIKGGNLRFAWKVLIVARRDKRMLDSSIYAKLILEL : 305
V. vinifera     : KVPFSLEKFTGLASRSVIEALARGFIRRGSLSLAWKLLLVAKDSKRMLGPSIYAKLIFEL : 333
Z. mays         : RVESALEQCVPTASRAVLEAMASGFIRAGKVDRVRKLLELARINNRTLHPSIYVKLMLEA : 320
O. sativa       : KMEDALEQCVPSASRAVLEAMVSGFIRAGKVGLARKLLEFATINKRTLSPSVHVKLMLEA : 302
A. coerulea     : KMPFDLEKFTVQASRSVIEAMARGFIRGGSFSLARKLLMIARDNNRKLDPSIHAKFILEL : 322
A. trichopoda   : KNAFNFADIMSSASRTVIEAMAKGCIRGGQFNLARKILLVAKDTGRTLDVSVSVMLLAEI : 354
T. plicata      : KTSFDLKITLASCNRNTLEAIARGFIKSGRCRQAREVLLAARDSLFKLDDGIHAKLIAQA : 312
C. richardii    : GIAWKLLYSHAPSSLLAFEALCKGHIANGLLYRALYAKQKAENSGMIIGQGLYSELVLLA : 214
D. complanatum  : HSAIDLYSQIEGASSSTTETLAMSLAKVDMFEQAVEVVKSKHLASSQETASVYVKLMVAA : 279
M. polymorpha   : GVAMQLHRGVGQDSIRAAQTLASALAKCGRLEEAMEVAKNLLDSGRKVDASVYRTITEWA : 400

                      *        440          *        460          *        480
A. thaliana     : AKNPDKYHLVVALLEELKKREDLKLSQQDCTSIMKICVKLGEFELVESLFDWFKASNREP : 361
P. trichocarpa  : GKNPDKHVLAEALLDELAEREDLNLSQQDCTAVMKVCIKLGKFEAVESLFNWFRQSGHEP : 372
G. raimondii    : GKNPDKHILVEELLGDLGERDDLNLSQQDCTAIMKVCIRHRKFEIVESLFYWFKQSGRDP : 412
G. max          : GKNPDRHRHVLPLLDELGERDELNLSQQDCTAIMKVCVKMGKFEVVESLFSWFKQSGYQP : 365
V. vinifera     : GKNPDKHSLVQALLDELGEREDLKLSHQDCTAVMKVCIRLGKFEIVESLFNWYKQSENSP : 393
Z. mays         : TRTPEGYGLASALVDELGERPELELRPQDCTAVMKVCIRLRRYAAVESLFSWFRGAIGSP : 380
O. sativa       : VRTPEGYGLAAALLDELGERPELHVRQQDCTAVMKVCVKLRRYAAVESLFGWFRDTGGRP : 362
A. coerulea     : VKNPDKYKLVSTLLTELAEMEEMNLSQQDCTAVMKVCIRLGKFEIVEILYNWFKESGRDI : 382
A. trichopoda   : AKFPDKKKLAIRILEELGEREELNLRQQDSTAIMKACVKLQRFDAVESLYNWFMQSGQDL : 414
T. plicata      : SKSVGWHNLAGKLIEELGMRDELKLEIQDCTAVMKGCIRLGMYEAVESLFFWWKESGHRP : 372
C. richardii    : AR-LNKVKEMKLLVEELGSFGELQLGLEHCTSIMAACRKSHMHDAVLSLFEWWKQAGFLP : 273
D. complanatum  : GSLRMH-KVVRVLLEELTLQN-LHLRLEDCTAVMALCSKLKMHDEAERLYSEYLQAGLQP : 337
M. polymorpha   : CRTGSR-NLVTRLWQNVNLSN-LNFKLQDYTSLMASCSKLGLYEIIDRLYQDFLDSGLEP : 458

                      *        500          *        520          *        540
A. thaliana     : SVVMYTTMIHSRYSEQKYREAMSVVWEME-ESNCLLDLPAYRVVIKLFVALDDLGRAMRY : 420
P. trichocarpa  : SVVMYTTLIHSRYSESKYREALAVVWEME-GSDCLFDLTAYRVVIKLFVALNDLPRAVRY : 431
G. raimondii    : SVVMYTTLLHSRYSENKYMEALALVWEME-ARECLLDLPAYRVVIKLFIGLKDLARAVRY : 471
G. max          : SIVMFTSVIHSRYTEKKYREALAVVWEME-ASNCLFDLPAYRVVIKLFVALNDLSRATRY : 424
V. vinifera     : SVVMYTTLIHSRYTEKKYREALAVVWEME-ASDCVFDLPAYRVVIKLFIALNDLSRTGRY : 452
Z. mays         : TVVMYTTVIHSRCRDGRHREALSLVWEME-QAGCLLDLPAYRVIVKLCVALHDPGRALRY : 439
O. sativa       : TVVMYTAVIHSRCRDGRHREALSLAWEMERHAGGLLDLPAYRVLVKLCVALRDHERGVRY : 422
A. coerulea     : SVVMYTTLIHSRYCENNYREALAVVWEME-QSNCLFDLPAYRVVIKLFVALNDISRAVRY : 441
A. trichopoda   : SVVMYTTVVHSRYIGKRYREAMALVWEME-GSNCLLDLPAYRVIIRLCVALDDLARAVRY : 473
T. plicata      : NVVMYTTIMHSRYCSSKFREAFALVWEME-ESNCVLDLPAYRVIIRLCAELDDLSRAARY : 431
C. richardii    : NVVMYQIVMTSLLDMGKPREALAIYWEMG-KRGFAPHLPVYTALIDICVELKDVHRALRM : 332
D. complanatum  : NIVMYTVVMKARSRAGKDREAMAVYWEIQ-NRGLVLDLVAYQVIMSVLGRLGDVSRAAKL : 396
M. polymorpha   : NIVMYTTLLSVLSREGRYREAVALIWEME-EVGCEPDLMAYEVMLDVCAKLEDLNRAMKV : 517
```

```
                        *       560       *       580       *       600
A. thaliana     : YSKLKEAGFSPTYDIYRDMISVYTASGRLTKCKEICKEVEDAGLRLDKDTSFRLLQLEKQ : 480
P. trichocarpa  : FSKLKEAGLSPTYDIYRNLITLYMVSGRLAKCKEVWKEAEMAGFKFSKEMAAGLLQLKRE : 491
G. raimondii    : FSKLKEAGFSPTYDMYRDLINMYMVAGRVGKCKEVCKEASMAGFSLDKRTLLNLSKLEKD : 531
G. max          : FSKLKEAGFSPSFGLYKDMLQIYMASGRIAKCKELCREAEIAGFKLDKYLVSVR------ : 478
V. vinifera     : FSKLKEAGFSPTYDIYRDMLKIYMVFRRLAKCREVCKELEMSGFKLDKGTLSQLLQLEKE : 512
Z. mays         : LSRMKEAGFIPTGDMYDSLIEGYLADGRLAKCRQLIRDAESAGVKLDRRLLSRLSETGGR : 499
O. sativa       : LARMKDAGFVPTGDMYGGLIGGYAAEGRMGRCRRLIREAELAGVKLERRLLSRLSEMGVE : 482
A. coerulea     : FSRLKEAGFTPTYDVYRDLVTIYAASGRLAKCKEVCKEVEMAGFKLDDQTASLLLEMGIE : 501
A. trichopoda   : FSRLKDAGFSPTHDIYSDLIELYARRGRLAKCREIRKEMEMVGFKLGAKIVALLEGDGKV : 533
T. plicata      : FSKMKDASFVPTRDIYTNLIVLYFKSGRVVKCRELLKEMERIGIKPNLEILRQRFGILSE : 491
C. richardii    : LSHMKESNITPTGNIYKKLIKLCNQDGRFGKAKELAEAMRKEGLPIHDVEAHTDLCF--- : 389
D. complanatum  : FLEMKHNNFSPTPDICNTLLKLYFKDGRFGKAKRILQEMQIMGYVADSEVVSLVKQNAWK : 456
M. polymorpa    : YKDLKNAGYIPTPDFYNTLIQLFVKKGSLSRAKDMTREMSSRGYTPSAETLSYLSAAAA- : 576

                        *       620
A. thaliana     : TMSLLH-------------- : 486
P. trichocarpa  : TRGSPALHLKAHEGQEKPLE : 511
G. raimondii    : ILG----------------- : 534
G. max          : -------------------- :   -
V. vinifera     : NR------------------ : 514
Z. mays         : HP------------------ : 501
O. sativa       : HSQL---------------- : 486
A. coerulea     : DRYAM--------------- : 506
A. trichopoda   : KLIAVSSSKDDRVSEGNLTC : 553
T. plicata      : TSAL---------------- : 495
C. richardii    : -------------------- :   -
D. complanatum  : H------------------- : 457
M. polymorpa    : -------------------- :   -
```

Supplementary Figure S2. Motifs and Sequence analysis of RFCD1. Amino acid sequence alignment of RFCD1 from various species. Strictly conserved amino acids are indicated in black, and closely relatedly residues are indicated in grey. The sequence alignment was constructed using MEGA7 software. *A. thaliana*, *Arabidopsis thaliana*. *P. trichocarpa, Populus trichocarpa. G. raimondii, Gossypium raimondii. G. max, Glycine max. V. vinifera, Vitis vinifera. Z. mays,Zea mays. O. sativa, Oryza sativa. A. coerulea, Arundina coerulea. A. trichopoda, Arundina trichopoda. T. plicata, Thuja plicata. C. richardii, Cyathea richardii. D. complanatum*, *Davallia complanatum. M. polymorpa, Marchantia polymorpha.*

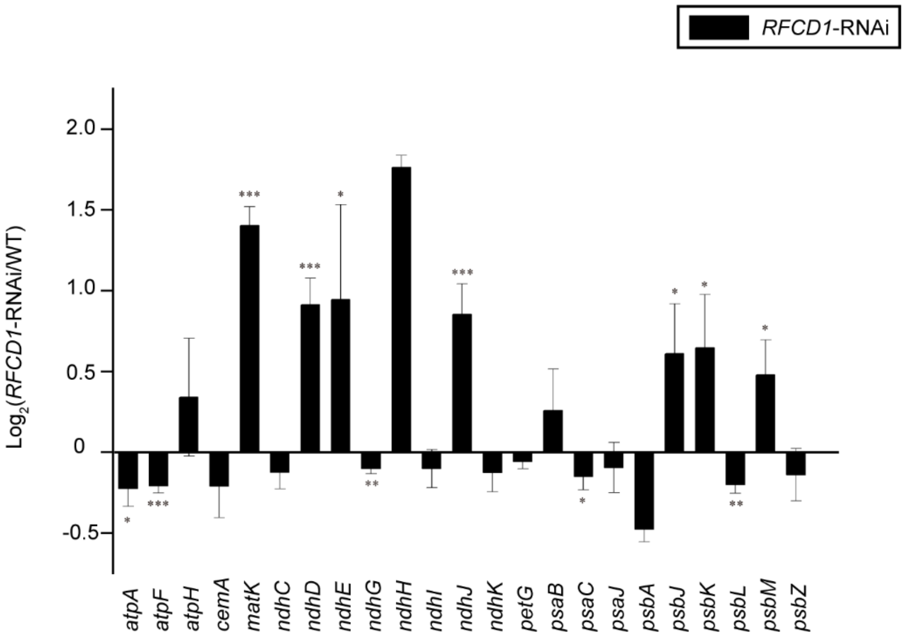

Supplementary Figure S3. Transcript levels of chloroplast genes that transcribed by unknown type of RNA polymerase. Data (means ± SE; n = 3 independent biological replicates) are given as $\log_2$ of *RFCD1*-RNAi-1/wild-type ratios. RNA was extracted from 7-day-old seedlings. ***$P < 0.001$, **$P < 0.01$, *$P < 0.05$, by Student's t-test.

A

Sample ID	Raw reads	Raw bases	Clean reads	Clean Bases	Error%	Q20%	Q30%	GC%
WT_1	47618242	7190354542	47215108	7104525517	0.0247	98.14	94.51	45.63
WT_2	45814624	6918008224	45429826	6839011724	0.0248	98.08	94.35	45.9
WT_4	41270346	6231822246	40927384	6163468023	0.0249	98.04	94.22	45.7
RFCD1-RNAi-1_1	45373936	6851464336	44836582	6747001880	0.0254	97.89	93.8	45.35
RFCD1-RNAi-1_2	45017706	6797673606	44662430	6718752303	0.0247	98.11	94.43	45.39
RFCD1-RNAi-1_3	51705130	7807474630	51293706	7713417763	0.0248	98.11	94.4	45.51

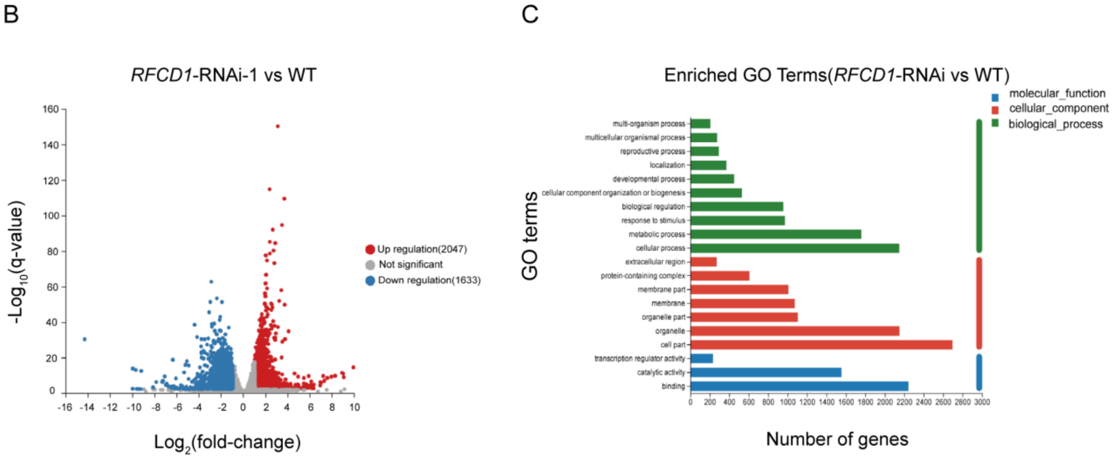

Supplementary Figure S4. Transcriptome analysis of WT and *RFCD1*-RNAi-1 plants.

(A) Summary of the transcriptome data of wild-type *Arabidopsis thaliana* and *RFCD1*-RNAi-1 plants. 'Q20' represents nucleotides with quality values larger than 20 in reads. 'Q30' represents nucleotides with quality values larger than 30 in reads. (B) Volcano plot showing differences in gene expression in *RFCD1*-RNAi-1 plants plotted against -$\log_{10}$ (q-value) highlighting WT plants (q-value < 0.05, n = 3 biological replicates, ANOVA). ANOVA is a statistical method used to determine if there are significant differences between the means of three or more independent groups. A q-value is a p-value that has been adjusted for the false discovery rate (FDR, the proportion of false positives). (C) GO enriched pathways for DEGs. The enriched molecular function terms of DEGs among WT and *RFCD1*-RNAi-1 lines. GO, Gene Ontology. DEG, Differential Gene Expression.

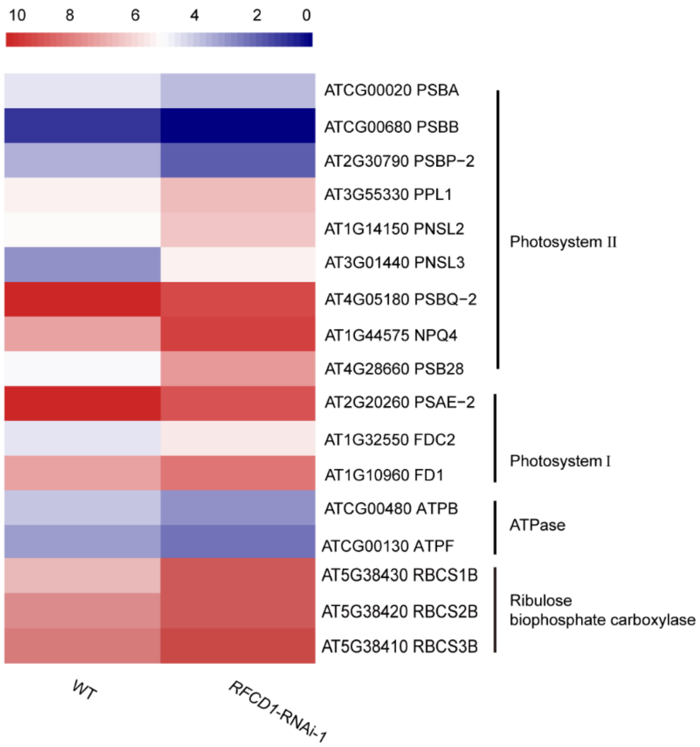

Supplementary Figure S5. Heatmap of the DEGs enriched in photosynthesis proteins. The bar represents the scale of the expression levels for each gene (log_2 FPKM) in WT and *RFCD1*-RNAi-1 lines as indicated by rectangles (p-adjust ≤ 0.05). FPKM represents fragments per kilobase of transcript per million mapped reads. The details of each gene are presented in Table S3.
