# Supplementary material for: A PPR Protein RFCD1 Affects Chloroplast Gene Expression and Chloroplast Development in Arabidopsis
Source: Plants (Basel). 2025 Mar 15;14(6):921. doi: 10.3390/plants14060921 (PMC11944589; doi:10.3390/plants14060921)
Supplement: Supplementary file 1 [file plants-14-00921-s001.zip › Table S4.pdf]

Table S4. List of primers used in this study.

Primer name	Sequences (5’-3’)	Purpose
psbA-F:<br>psbA-R:	ATGACTGCAATTTTAGAGAGACGCG<br>TTATCCATTTGTAGATGGAGCCTCA	RNA blot
16S-F:<br>16S-R:	AACGGCTGCTAATACCCCGTAGGCT<br>TCCAGTCACTAGCCCTGCCTTCGGC	RNA blot
23S-F:<br>23S-R:	AGGAGAGCACTCATCTTGGGGTGGG<br>TTCAAACGAGGAAAGGCTTACGGTG	RNA blot
RFCD1-RNAi-F1:<br>*RFCD1*-RNAi-R1:	CTTTCCATGGTGTCTCTTTGTCACTTA<br>CTTCATTTAAATGGTTTATCGGGTTTTTG	RNAi vector
RFCD1-RNAi-F2:<br>*RFCD1*-RNAi-R2:	CCTTTCTAGATGTCTCTTTGTCACTTA<br>CTTTGGATCCGGTTTATCGGGTTTTTG	RNAi vector
RFCD1-RTPCR-F:<br>*RFCD1*-RTPCR-R:	GCTATGATCAAGGGATGCAT<br>CTCTGTTTGATGCTTTAAAC	RT-PCR
RFCD1-60AA-GFP-F:<br><br>*RFCD1*-60AA-GFP-R:	ATTTGGAGAGGACAGCCCAAGCTTATGATAC TACACTGTCCTGTCTC<br>CTTGCTCACCATGGATCCGTCGACATTAACA CCAAAATCAGGAGGAAG	Overexpression vector
ndhA-F:<br>*ndhA*-R:	TTGACGCCACAAATTCCAT<br>TTAGGTGGTCTGCGAGCTG	quantitative RT-PCR
petB-F:<br>*petB*-R:	ATTGGGCGGTCAAAATTGTA<br>AGACGGCCGTAAGAAGAGGT	quantitative RT-PCR
petD-F:<br>*petD*-R:	TCCTTTTGCAACTCCTTTGG<br>CCGCTGGTACTGAAACCATT	quantitative RT-PCR
psaA-F:<br>*psaA*-R:	GCCAAGAAATCCTGAATGGA<br>CATCTTGGAACCAAGCCAAT	quantitative RT-PCR
psbA-F:<br>*psbA*-R:	GAGCAGCAATGAATGCGATA<br>CCTATGGGGTCGCTTCTGTA	quantitative RT-PCR
psbC-F:<br>*psbC*-R:	ACTTCCCCACCTAGCCACTT<br>AGCCCAAAACTGCAGAAGAA	quantitative RT-PCR
psbD-F:<br>*psbD*-R:	CACAAATCTTTGGGGTTGCT<br>CCATCCAAGCACGAATACCT	quantitative RT-PCR
psbF-F:<br>*psbF*-R:	GGACCTATCCAATTTTTACAGTGC<br>GTTGGATGAACTGCATTGCT	quantitative RT-PCR
psbH-F:<br>*psbH*-R:	TCTAGATCTGGTCCAAGAAGCA<br>CATTGCAACACCCATCAAAG	quantitative RT-PCR
psbN-F:<br>*psbN*-R:	GGAAACAGCAACCCTAGTCG<br>CGTGTTCCTCGAATGGATCT	quantitative RT-PCR
psbI-F:<br>*psbI*-R:	TTTCTCTCTTCATATTTGGATTCCT<br>TTCTTCACGTCCCGGATTAC	quantitative RT-PCR
rbcL-F:<br>*rbcL*-R:	GTGTTGGGTTCAAAGCTGGT<br>CATCGGTCCACACAGTTGTC	quantitative RT-PCR
atpB-F:	CCGTTTCGTACAAGCAGGAT	quantitative

atpB-R:	CGGGGTCAGTCAAATCATCT	RT-PCR
atpE-F:<br>*atpE*-R:	TCCACAAGAAGCTCAGCAAA<br>GTGTCCGAGCTCGTCTGAG	quantitative<br>RT-PCR
atpI-F:<br>*atpI*-R:	ATTGGCAAATAGGGGGTTTC<br>GCCGTCAGTTGGAATTGTTT	quantitative<br>RT-PCR
ndhB-F:<br>*ndhB*-R:	CCAGAAGAAGATGCCATTCA<br>TCATCAATGGACTCCTGACG	quantitative<br>RT-PCR
ndhF-F:<br>*ndhF*-R:	CGGCGGGTATTTTTCTTGTA<br>GGCTAAACCCCGCTTAATGT	quantitative<br>RT-PCR
psaJ-F:<br>*psaJ*-R:	ATGGTTCGGTTCGTTAGCAG<br>GGGAAATGTTAATGCATCTGG	quantitative<br>RT-PCR
rps18-F:<br>*rps18*-R:	CAAGCGATCTTTTCGTAGGC<br>AAAGTCACTCTATTCACCCGTCT	quantitative<br>RT-PCR
accD-F:<br>*accD*-R:	TGTGGATTCAATGCGACAAT<br>TTTTGCGCAGAGTCAATACG	quantitative<br>RT-PCR
rpoA-F:<br>*rpoA*-R:	GCGATGCGAAGAGCTTTACT<br>CCAGGACCTTGGACACAAAT	quantitative<br>RT-PCR
rpoB-F:<br>*rpoB*-R:	AAAAAGCACGGATACGGATG<br>CTTCTTGAATGCCCCGATTA	quantitative<br>RT-PCR
rpoC1-F:<br>*rpoC1*-R:	TCGGATACGAAGATATCAAATGG<br>TTAGTTATGGGCCTAGCAAAAGA	quantitative<br>RT-PCR
rpoC2-F:<br>*rpoC2*-R:	ATGGAGCCCGTAAAGGAGTT<br>CGTCTGCTAAGACACGACCA	quantitative<br>RT-PCR
ycf2-F:<br>*ycf2*-R:	TAGCCCTCGGTCTATTGGTG<br>GGATCCACTTTTTGGGGAAT	quantitative<br>RT-PCR
atpA-F:<br>*atpA*-R:	CGGAAATCTTACCTCGACCA<br>ATGGGTGACGGTTTGATGAT	quantitative<br>RT-PCR
atpF-F:<br>*atpF*-R:	GCTCCTTCACGCAGTTCTTC<br>TACTTGGGTCACTGGCCATC	quantitative<br>RT-PCR
atpH-F:<br>*atpH*-R:	ATCCACTGGTTTCTGCTGCT<br>TTCCTTCTGCCTCAGGTTGT	quantitative<br>RT-PCR
cemA-F:<br>*cemA*-R:	TTTGCCCTGGTTGATCTCTC<br>TTGGATCGTTTCTTTGTGGA	quantitative<br>RT-PCR
matK-F:<br>*matK*-R:	ATCCTTTGTTGCCAGAATGC<br>TTTTTCTACGCAAGCGGTCT	quantitative<br>RT-PCR
ndhC-F:<br>*ndhC*-R:	TATAGAACCGATCGGGGATG<br>AACTCATTGCCCACGGATAC	quantitative<br>RT-PCR
ndhD-F:<br>*ndhD*-R:	TGGAGAATGGGAATAGATGGAC<br>TCCCGAGAAGAAAATGATCCTA	quantitative<br>RT-PCR
ndhE-F:<br>*ndhE*-R:	TGGATTGATCACAAGTCGAAA<br>AGCGGCTGCAATTGCTATAA	quantitative<br>RT-PCR
ndhG-F:<br>*ndhG*-R:	TTGCCTGGACCAATACATGA<br>ACATTTATGGCCCCCACATA	quantitative<br>RT-PCR

ndhH-F:<br>*ndhH*-R:	ATGGGAAATTCAATGGCAAA<br>TCAAAGCCCCTGCTTTCTAA	quantitative<br>RT-PCR
ndhI-F:<br>*ndhI*-R:	TTTGCCTGTTGTTGATTGGA<br>ATTGGTAAACGACCCAAAGC	quantitative<br>RT-PCR
ndhJ-F:<br>*ndhJ*-R:	CGTTTTCTGGGTTTGGAAAA<br>AGGCCACCCTATCCAACTCT	quantitative<br>RT-PCR
ndhK-F:<br>*ndhK*-R:	GCAGTCCGCATATTGGAAAT<br>CGTGGGACGATACTGGACTT	quantitative<br>RT-PCR
petA-F:<br>*petA*-R:	CAGAGGGCGAATCCATTAAA<br>GCCAAAACAACCGATCCTAA	quantitative<br>RT-PCR
petG-F:<br>*petG*-R:	TCTAATTCCTATTACTTTGGCTGGA<br>CCAACTGATCACCACGTCTG	quantitative<br>RT-PCR
psaB-F:<br>*psaB*-R:	GGACCCCACTACTCGTCGTA<br>ATTGCTAATTGCCCGAAATG	quantitative<br>RT-PCR
psaC-F:<br>*psaC*-R:	GAGCATGCCCTACAGACGTA<br>CAGGCGGATTCACATCTCTT	quantitative<br>RT-PCR
psaI-F:<br>*psaI*-R:	ACTTACCCTCTATTTTTGTGCCTTT<br>TGAATATGAAGAAATAAAGAAGCCATT	quantitative<br>RT-PCR
psbJ-F:<br>*psbJ*-R:	CTGGAAGGATTCCTCTTTGG<br>CAGGGATGAACCTAATCCTGA	quantitative<br>RT-PCR
psbK-F:<br>*psbK*-R:	AGGCCTACGCCTTTTTGAAT<br>CGAAAACTTACAGCGGCTTG	quantitative<br>RT-PCR
psbL-F:<br>*psbL*-R:	CAATCAAATCCGAACGAACA<br>GAAATAATTCGAAAATAAAACAGCAA	quantitative<br>RT-PCR
psbM-F:<br>*psbM*-R:	TGCACTCTTCATTCTCGTTCC<br>TCATTTTGACTAACGGTTTTTACG	quantitative<br>RT-PCR
psbZ-F:<br>*psbZ*-R:	TGCTTTCCAATTGGCAGTTT<br>GTTACTCGACCAACCATCAGG	quantitative<br>RT-PCR
ycf15-F:<br>*ycf15*-R:	GCGAACAACCGGAGCTATTA<br>CCGACATGCGTATTTTTGATT	quantitative<br>RT-PCR
ycf3-F:<br>*ycf3*-R:	TCCAATACTCAGCGGCTTG<br>TTCGGGCATTAGAACGAAAC	quantitative<br>RT-PCR
ycf4-F:<br>*ycf4*-R:	TTTCTATGGGATCGCAGGTC<br>GGAAATCCCCAACGAAAAAT	quantitative<br>RT-PCR
